# Artificial Intelligence for Hip Fracture Detection and Outcome Prediction: A Systematic Review and Meta-analysis

**DOI:** 10.1001/jamanetworkopen.2023.3391

**Published:** 2023-03-17

**Authors:** Johnathan R. Lex, Joseph Di Michele, Robert Koucheki, Daniel Pincus, Cari Whyne, Bheeshma Ravi

**Affiliations:** 1 Division of Orthopaedic Surgery, Department of Surgery, University of Toronto, Toronto, Ontario, Canada; 2 Institute of Biomedical Engineering, University of Toronto, Toronto, Ontario, Canada; 3 Orthopaedics Biomechanics Laboratory, Sunnybrook Research Institute, Toronto, Ontario, Canada; 4 Temerty Faculty of Medicine, University of Toronto, Toronto, Ontario, Canada; 5 Division of Orthopaedic Surgery, Sunnybrook Health Sciences Centre, Toronto, Ontario, Canada

## Abstract

**Importance:**

Artificial intelligence (AI) enables powerful models for establishment of clinical diagnostic and prognostic tools for hip fractures; however the performance and potential impact of these newly developed algorithms are currently unknown.

**Objective:**

To evaluate the performance of AI algorithms designed to diagnose hip fractures on radiographs and predict postoperative clinical outcomes following hip fracture surgery relative to current practices.

**Data Sources:**

A systematic review of the literature was performed using the MEDLINE, Embase, and Cochrane Library databases for all articles published from database inception to January 23, 2023. A manual reference search of included articles was also undertaken to identify any additional relevant articles.

**Study Selection:**

Studies developing machine learning (ML) models for the diagnosis of hip fractures from hip or pelvic radiographs or to predict any postoperative patient outcome following hip fracture surgery were included.

**Data Extraction and Synthesis:**

This study followed the Preferred Reporting Items for Systematic Reviews and Meta-analyses and was registered with PROSPERO. Eligible full-text articles were evaluated and relevant data extracted independently using a template data extraction form. For studies that predicted postoperative outcomes, the performance of traditional predictive statistical models, either multivariable logistic or linear regression, was recorded and compared with the performance of the best ML model on the same out-of-sample data set.

**Main Outcomes and Measures:**

Diagnostic accuracy of AI models was compared with the diagnostic accuracy of expert clinicians using odds ratios (ORs) with 95% CIs. Areas under the curve for postoperative outcome prediction between traditional statistical models (multivariable linear or logistic regression) and ML models were compared.

**Results:**

Of 39 studies that met all criteria and were included in this analysis, 18 (46.2%) used AI models to diagnose hip fractures on plain radiographs and 21 (53.8%) used AI models to predict patient outcomes following hip fracture surgery. A total of 39 598 plain radiographs and 714 939 hip fractures were used for training, validating, and testing ML models specific to diagnosis and postoperative outcome prediction, respectively. Mortality and length of hospital stay were the most predicted outcomes. On pooled data analysis, compared with clinicians, the OR for diagnostic error of ML models was 0.79 (95% CI, 0.48-1.31; *P* = .36; *I*^2^ = 60%) for hip fracture radiographs. For the ML models, the mean (SD) sensitivity was 89.3% (8.5%), specificity was 87.5% (9.9%), and F1 score was 0.90 (0.06). The mean area under the curve for mortality prediction was 0.84 with ML models compared with 0.79 for alternative controls (*P* = .09).

**Conclusions and Relevance:**

The findings of this systematic review and meta-analysis suggest that the potential applications of AI to aid with diagnosis from hip radiographs are promising. The performance of AI in diagnosing hip fractures was comparable with that of expert radiologists and surgeons. However, current implementations of AI for outcome prediction do not seem to provide substantial benefit over traditional multivariable predictive statistics.

## Key Points

**Question:**

For patients with hip fractures, how well do current artificial intelligence algorithms perform at diagnosing fractures and predicting postoperative outcomes?

**Findings:**

This systematic review and meta-analysis of 39 studies identified similar error rates of hip fracture diagnosis between artificial intelligence models and expert clinicians. There was minimal advantage of machine learning models over traditional regression techniques for postoperative outcome prediction.

**Meaning:**

These findings suggest that artificial intelligence has the potential to automate hip fracture diagnosis; however, complicated, uninterpretable models may not provide benefit over traditional, interpretable models for patient-specific outcome prediction.

## Teaser

This systematic review and meta-analysis evaluates the performance of AI algorithms compared with current practices to diagnose hip fractures and estimate postoperative clinical outcomes.

## Introduction

The number of artificial intelligence (AI) algorithms in the medical literature and health care industry is increasing rapidly. This increase is due to relatively recent advances in computational power, data accessibility, and model complexity through mathematical and computer science research.^1,2^ Correspondingly, there has been an increasing number of AI algorithms and AI-enabled medical devices approved by the US Food and Drug Administration (79 and 343, respectively).^3,4,5^ There are a large number of potential applications for AI; however, most of the models developed have focused on the interpretation of medical imaging and clinical decision support systems.^3^ Across the literature, AI models are beginning to show the ability to automate and potentially improve clinicians’ diagnostic and clinical decision-making.^6,7^ However, preceding research and literature reviews have predominantly been conducted in the fields of radiology, pathology, oncology, and ophthalmology, with a smaller proportion of research being conducted within the surgical specialties and particularly orthopedic surgery.^6,7,8,9,10^

The most prominent domain within orthopedic surgery in which AI research has been conducted is in hip fractures. Among elderly populations, hip fractures make up more than 14% of total fractures, although they represent a disproportionate 72% of fracture-related health care costs.^11,12^ Approximately 300 000 hip fractures occur per year in the US alone.^13,14^ Despite prevention efforts, this number is steadily increasing due to an aging population.^13,15,16^ Worldwide, this number is expected to reach 6.3 million hip fractures at a cost of $131.5 billion per year by 2050.^17,18^ In addition to their significant prevalence and economic impact, hip fractures are also associated with significant individual morbidity and mortality, with a 1-year mortality rate of approximately 25% to 30%.^19,20,21,22^ Therefore, technology to improve the efficiency of managing this condition has the potential to improve patient outcomes and provide economic benefit to health care systems.

Improvement of the efficiency of hip fracture diagnosis and surgery has received considerable attention in recent years. Expedited management and comprehensive care pathways have been proven to improve outcomes, including survival rate, for these patients.^22,23,24^ These circumstances provide an ideal use case for this novel technology should its performance be equal or superior to human performance. Image analysis and clinical decision support systems powered by AI may automate sections of the diagnostic pathway and improve outcome prediction accuracy.^6,7,8^ Expedited diagnosis by leveraging this technology would lead to rapid diagnosis and access to surgical care. Perioperative risk stratification for clinicians and hospitals caring for these patients can assist in decision-making, accurate expectation management, and financial and resource planning. Moreover, these applications have the potential to reduce errors secondary to physician fatigue from repetitive cognitive demands and improve informed decision-making for patients and families.^25,26,27^ In this systematic review, we sought to evaluate the literature and performance of AI algorithms designed to improve the management of hip fractures in elderly patients across 2 domains: (1) to evaluate the performance of AI compared with health care professionals for detection of hip fractures on medical imaging and (2) to determine the accuracy of AI at predicting various postoperative clinical outcomes compared with traditional statistical methods.

## Methods

### Search Strategy and Study Selection

A systematic review of the literature was performed using MEDLINE, Embase, and the Cochrane Library for all articles published from database inception to January 23, 2023. The Preferred Reporting Items for Systematic Reviews and Meta-analyses (PRISMA) 2020 reporting guideline was used to design the review.^28^ The inclusion criteria and analysis plan were decided a priori and registered on PROSPERO (CRD42022351255). In each database, the following keywords were combined to identify relevant articles: *hip* OR *neck of femur* OR *femoral neck* OR *intertrochanteric* OR *pertrochanteric* OR *subtrochanteric* AND *fracture* OR *broken* AND *artificial intelligence* OR *AI* OR *machine learning* OR *ML* OR *computer vision* OR *neural network*. A manual reference search of included articles was also undertaken to identify any additional relevant articles. This review had 2 groups: (1) studies that developed any machine learning (ML) or deep learning model for the diagnosis of hip fractures using medical imaging and (2) studies that developed a model designed to predict any postoperative patient outcome following hip fracture surgery.

### Eligibility Criteria and Data Extraction

Artificial intelligence models evaluating the radiographic diagnosis or outcome prediction of hip fractures, including femoral neck, intertrochanteric, and subtrochanteric fractures, were included, with isolated fractures of any other anatomical site being excluded (acetabular, pelvic, femoral head, midshaft femur, or distal femoral fractures). Studies designed to diagnose hip fractures from medical imaging were included if they were based on anteroposterior and lateral hip or anteroposterior pelvic plain radiographs. Ground truth must have been based on image review by a consensus medical expert group, radiologist report and image review by a staff radiologist, surgical confirmation, or cross-sectional imaging (computed tomography or magnetic resonance imaging) confirmation. All level 3 or higher studies, including randomized clinical trials, prospective studies, and retrospective studies, were included. Studies were not excluded based on the presence or absence of a comparator group or language of publication. Case reports, literature reviews, abstracts, unpublished studies, and nonhuman studies were excluded. Authors were contacted if needed to retrieve copies of unavailable manuscripts.

Screening of search results based on titles and abstracts was performed by 2 independent reviewers (J.D.M. and R.K.), with conflicts resolved by inclusion of a third reviewer (J.R.L.). Three reviewers independently assessed the eligibility following abstract screening for study inclusion according to the inclusion and exclusion criteria. In cases of conflict, decisions were made through consensus agreement among the 3 reviewers. Eligible full-text articles were evaluated, and relevant data were extracted independently by 2 reviewers (J.D.M. and R.K.) using a template data extraction form, with conflicts resolved by inclusion of a third reviewer (J.R.L.).

When studies generated multiple AI models to predict the same outcome, we recorded performance data from the best-performing model on the test or out-of-sample data subset. This process ensured that only the best-performing model would be chosen for use in clinical practice. When reported, the accuracy of staff (consultant-level) orthopedic surgeons or radiologists at diagnosing hip fractures was compared with AI models. Performance of resident physicians was not included. For the comparator groups evaluating clinician performance, if multiple clinicians were evaluated, their mean accuracy score was calculated from their performance on the same out-of-sample data subset. A mean score was calculated for clinician performance because this most closely resembles the diagnostic accuracy of the current workflow, with different radiologists or surgeons making the diagnosis, depending on the day.

For studies that predicted postoperative outcomes, the performance of traditional predictive statistical models, either multivariable logistic or linear regression, was recorded and compared with the performance of the best ML model on the same out-of-sample data set. This method of statistical analysis is most typically used to generate predictive or prognostic clinical models and was therefore used as a baseline performance indicator. Any more advanced algorithm type, including regression with regularization, was considered a form of ML.

### Statistical Analysis

Odds ratios (ORs) with 95% CIs were used for dichotomous outcome measures. Heterogeneity was assessed using the *I*^2^ statistic, with *I*^2^ ≥ 75% indicating considerable heterogeneity. A random-effects model to pool the data was planned to be used if considerable heterogeneity was found (*I*^2^ ≥ 50%); otherwise, a fixed-effect model and a Mantel-Haenszel statistical method were used. Sensitivity and specificity of the diagnostic AI model’s performance were plotted and compared with the performance of medical experts, with a pooled 95% CI around the mean. Youden index scores were calculated from sensitivity and specificity when reported. The area under the curve (AUC) of each predictive statistical and AI model were compared using a 2-tailed, unpaired *t* test. Microsoft Excel (Microsoft Corp) was used to extract data. For pooled data analysis, Review Manager (RevMan), version 5.4 (The Cochrane Collaboration) and Stata software, version 16.1 (StataCorp LLC) were used.

## Results

### Study Selection

Of 39 studies that met all criteria and were included in this analysis, 18 studies^29,30,31,32,33,34,35,36,37,38,39,40,41,42,43,44,45,46^ (46.2%) used AI models to diagnose hip fractures on plain radiographs and 21 studies^47,48,49,50,51,52,53,54,55,56,57,58,59,60,61,62,63,64,65,66,67^ (53.8%) used AI models to predict patient outcomes following hip fracture surgery. A PRISMA flowchart of included studies is displayed in eFigure 1 in Supplement 1. The characteristics of the included studies are given in Table 1 (diagnostic studies) and Table 2 (outcome prediction studies). Diagnostic studies were published between 2019 and 2022 and used a total of 39 598 plain radiographs to train, validate, and test AI models (Table 1; eTable 1 in Supplement 1). Outcome prediction studies were published between 2004 and 2022. Mortality followed by length of hospital stay were the most commonly predicted outcomes, with other predicted outcomes of 30-day complications, living situation, postoperative delirium, and modified functional independence measure.^47,58,61^ A pooled total of 714 939 hip fractures were used for training, validating, and testing ML models specific to postoperative outcome prediction. All databases used for outcome prediction are listed in eTable 2 in Supplement 1.

**Table 1.** Included Studies on Application of Artificial Intelligence for Diagnosis of Hip Fractures

Source (country)	Input imaging	No. of output classes	Output	Algorithm used	No. of radiographs	Training size, %	Validation size, %	Testing size, No. or %	Ground truth
Cheng et al,^29^ 2019 (Taiwan)	AP pelvic radiograph	2	Fractured (femoral neck and trochanteric); nonfractured	DCNN	3605	80	20	100	Radiologist report or CT report with each image reviewed by trauma surgeon
Urakawa et al,^30^ 2019 (Japan)	AP proximal femoral radiograph	2	Fractured (intertrochanteric); nonfractured	CNN	3346	80	10	10%	Surgically confirmed
Adams et al,^31^ 2019 (Australia)	AP hip cropped from AP pelvic radiograph	2	Fractured (femoral neck); nonfractured	AlexNet DCNN; GoogLeNet DCNN	640	80	20	160	Surgically confirmed
Mawatari et al,^32^ 2020 (Japan)	Pelvic radiograph	2	Fractured (proximal femur fracture); nonfractured	DCNN	352	85.8	NR	14.2%	CT or MRI confirmed
Jiménez-Sánchez et al,^33^ 2020 (Germany, Spain, and France)	AP and lateral pelvic radiograph, images were cropped	2	Fractured; nonfractured	ResNet-50; AlexNet	1347	70	10	20%	Image review by group of experts (1 staff trauma surgeon, 1 staff radiologist, 1 senior trauma resident)
		3	AO type A fracture; AO type B fracture; nonfractured	ResNet-50; AlexNet					
Yu et al,^34^ 2020 (US)	AP hip radiograph	2	Fractured; nonfractured	DCNN	627	60	20	20%	Surgically confirmed or CT confirmed
Kitamura,^35^ 2020 (US)	Pelvic radiograph	2	Normal; abnormal	Densenet-121 architecture	7337	70	NR	30%	Radiologist report and image review by a staff radiologist
		8	Normal; anterior pelvis; posterior pelvis; pelvic ring; proximal femur; acetabular; femur/acetabular; nonfemoral						
Krogue et al,^36^ 2020 (US)	Hip and pelvic radiograph, hips were labeled via bounding boxes	6	Normal; displaced femoral neck fracture; nondisplaced femoral neck fracture; intertrochanteric fracture; previous open reduction and internal fixation; previous arthroplasty	DCNN (DenseNet)	1999	61.1	24.4	14.5%	Consensus by experts; CT, MRI, postoperative imaging in the event of uncertainty
		2	Fractured; nonfractured						
Yamada et al,^37^ 2020 (Japan)	AP and lateral pelvic radiograph	3	Femoral neck fractures; trochanteric fractures; nonfractured	CNN	2923	89.7	10.3	NR	Consensus by experts and CT
Mutasa et al,^38^ 2020 (US)	AP pelvic radiograph	2	Femoral neck fracture (any Garden fracture); normal	CNN	1063	Unclear	20	Unclear	Image review by a single staff radiologist
		3	Femoral neck fracture (Garden I/II fracture); femoral neck fracture (Garden III/IV fracture); normal						
Beyaz et al,^39^ 2020 (Turkey)	AP hip cropped from AP pelvic radiograph, various image sizes assessed	2	Fractured (femoral neck); nonfractured	CNN; GA	234	NR	NR	NR	Unclear
Açıcı et al,^40^ 2021 (Turkey)	AP pelvic radiograph	2	Fractured (femoral neck); nonfractured	CNN; GA; PSO; LSTM; BILSTM	64	NR	NR	NR	Unclear
Bae et al,^41^ 2021 (Korea)	AP pelvic radiograph	3	Displaced fracture; nondisplaced fracture; nonfractured	CNN; ResNet 18 with CBAM	4189	80	10	10%	CT or MRI confirmed
Cheng et al,^42^ 2021 (Taiwan)	AP pelvic radiograph	3	Hip fracture only; pelvic fracture only; no acute finding	PelviXNet (DenseNets + FPN); CNN	5204	100	NR	1888	Image review by group of clinicians
Guy et al,^43^ 2021 (France)	AP and lateral pelvic radiograph	3	Femoral neck fracture; trochanteric fracture; nonfractured	Tensorflow deep learning algorithm	1309	80	10	10%	Unclear
Twinprai et al,^44^ 2022 (Thailand)	AP hip and pelvic radiograph	2	Fractured; nonfractured	DCNN	1000	90	NR	10%	Consensus by experts and CT/MRI
		3	No fracture; trochanteric; intracapsular						
Murphy et al,^45^ 2022 (UK)	AP pelvic radiograph	4	Normal; femoral neck fracture; intertrochanteric fracture; subtrochanteric fracture	CNN	3659	60	20	20%	Consensus by experts
Liu et al,^46^ 2022 (China)	AP hip radiograph	2	Nonfractured; fractured (intertrochanteric)	Faster RCNN	700	91.9	NR	8.1%	Unclear

Abbreviations: AO, atlanto-occipital; AP, anteroposterior; BILSTM, bidirectional long short-term memory; CBAM, convolutional block attention module; CNN, convolutional neural network; CT, computed tomography; DCNN, deep convolutional neural network; FPN, feature pyramid network; GA, general algorithm; LSTM, long short-term memory; MRI, magnetic resonance imaging; NR, not reported; PSO, particle swarm optimization; RCNN, region-based convolutional neural network.

**Table 2.** Studies on Application of Machine Learning Models for Predicting Postoperative Outcomes Following Hip Fracture Surgery

Source (country)	Algorithm used	Outcome predicted	No. of features in model	Time points of outcome	No. of output classes	Output	No. of hip fractures	Training size, %	Testing size, %	Ground truth
Ottenbacher et al,^47^ 2004 (US)	ANN	Living setting	13	80-180 d After discharge	2	Home; not at home	3708	66.60	33.40	Retrospective database review
Sund et al,^48^ 2009 (Finland)	Bayesian nonparametric MLP	Length of stay	22	NR	NR	NR	15 544	NR	NR	NR
Lin et al,^49^ 2010 (Taiwan)	ANN	Mortality	12	1 y	2	Die; survive	286	68.88	31.12	Retrospective database review
Shi et al,^50^ 2013 (China)	ANN	Mortality	8	1 y	2	Die; survive	2150	66.60	33.40	Retrospective database review
Karnuta et al,^51^ 2019 (US, UK)	NB	Length of stay; cost	7	NR	LOS: 3 classes	LOS: 1-3 d; 4-6 d; 7-9 d; ≥10 d	98 562	90	10	Retrospective database review
					Cost: 4 classes	Cost:<$8464; $8464-$26 313;>$26 313				
Chen et al,^52^ 2020 (Taiwan)	ANN	Mortality	9	Unclear (has stated both 30 or 90 d)	2	Die; survive	10 534	70.00	15.00	Retrospective database review
Zhang et al,^54^ 2020 (China)	BBN	Mortality	16	1 y	2	Die; survive	448	90	10	Retrospective database review
DeBaun et al,^53^ 2021 (US)	ANN	Mortality	47	30 d	2	30-d Mortality; survive	19 835	80	20	Retrospective database review
Cao et al,^55^ 2021 (Sweden)	CNN	Mortality	6	30 d	2	Die; survive	134 915	80	20	Retrospective database review
Cowling et al,^56^ 2021 (UK)	XGBoost algorithm, for tree models	Mortality	5	1 y	2	Die; survive	169 646	NR	NR	Retrospective database review
Forssten et al,^57^ 2021 (Sweden, US)	SVM; NB; RF	Mortality	30	1 y	2	Die; survive	124 707	80	20	Retrospective database review
Oosterhoff et al,^58^ 2021 (US, the Netherlands)	NN; SGM; SVM; RF; PLR	Postoperative delirium	6	30 d	2	Delirium; no delirium	28 207	80	20	Retrospective database review
Li et al,^59^ 2021 (China)	RF	Mortality	10	1 mo; 3 mo; 6 mo; 1 y; 2 y	2	Die; survive	1330	NR	NR	Unclear
Cary et al,^60^ 2021 (US)	MLP	Mortality	14	30 d; 1 y	2	Die; survive	17 140	Unclear	Unclear	Retrospective database review
Shtar et al,^61^ 2021 (Israel)	AdaBoost; CatBoost; ExtraTrees; KNN; RF; SVM; XGBoost	Motor functional independence measure	25	NR	2	Better than the median score; worse than the median score	1625	Unclear	Unclear	Retrospective database review
Zhong et al,^62^ 2021 (China)	PCR; SVM; BP	Length of stay	6	NR	NR	Outputting a specific LOS	182	80	20	Retrospective database review
Xing et al,^63^ 2022 (China)	RF; lasso regression	Mortality	7	1 y	2	Die; survive	591	70	30	Retrospective database review
Harris et al,^64^ 2022 (US)	Lasso regression	Mortality; major complications	15	30 d	NR	Outputting a specific risk	82 168	90	10	Retrospective database review
Oosterhoff et al,^65^ 2022 (the Netherlands, US)	SGB; RF; SVM; NN; Elastic−Net Penalized Logistic Regression	Mortality	10	90 d; 2 y	NR	Outputting a specific risk	2478	80	20	Retrospective database review
Kitcharanant et al,^67^ 2022 (Thailand)	GB; RF; ANN; LR; NB; SVM; KNN	Mortality	15	1 y	NR	Outputting a specific risk	492	70	30	Retrospective database review
Lei et al,^66^ 2023 (China)	RF; GBM; DT; eXGBoost	In-hospital mortality	6	NR	NR	Outputting a specific risk	391	66.60	165 External validation	Retrospective database review

Abbreviations: ANN, artificial neural network; BBN, bayesian belief network; BP, backpropagation; CNN, convolutional neural network; DT, decision tree; GB, gradient boosting; GBM, gradient boosting machine; KNN, K-nearest neighbor; LOS, length of stay; LR, linear regression; MLP, multilayer perceptron; NB, naive Bayes; NN, neural network; NR, not reported; PCR, principal component regression; PLR, penalized logistic regression; RF, random forest; SGB, stochastic gradient boosting; SGM, semiglobal matching; SVM, support vector machine.

### Hip Fracture Diagnosis

**Figure 1.**
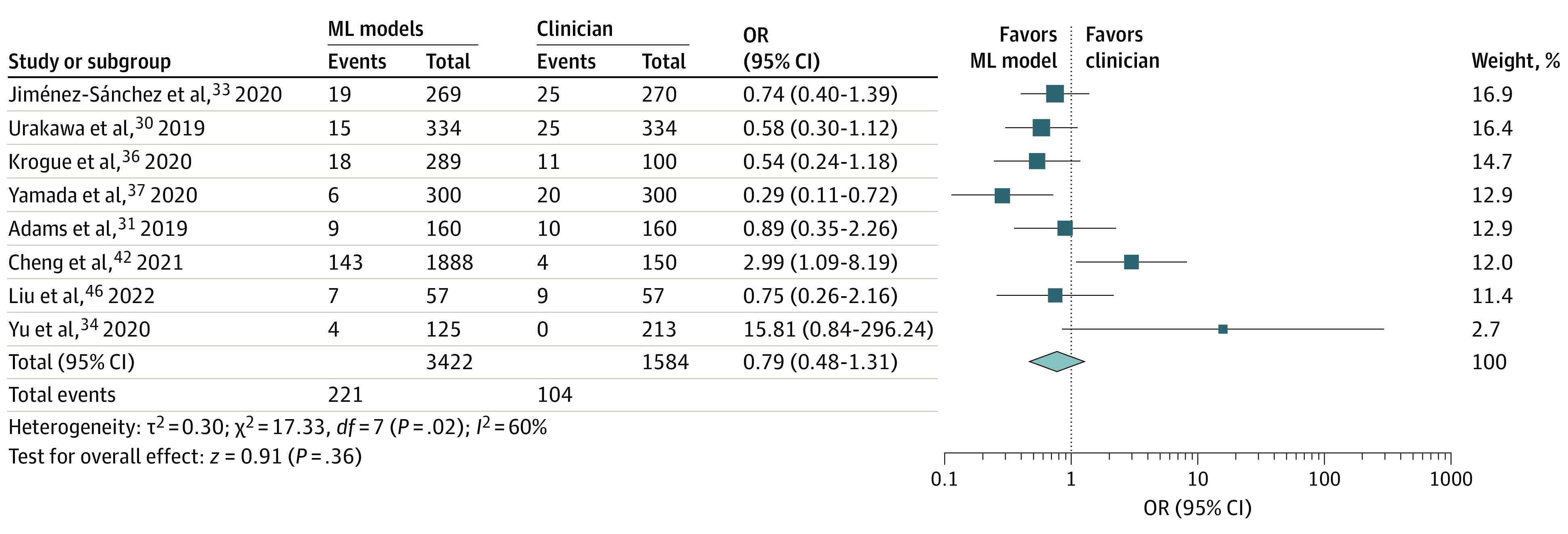
Forest Plot Demonstrating the Accuracy of Artificial Intelligence Models Compared With Clinicians in Diagnosing Hip Fractures ML indicates machine learning; OR, odds ratio.

All included studies developed a form of convolutional neural network model to diagnose fractures (Table 1). Comparative quantitative data for accuracy of hip fracture diagnosis as per plain radiographs were available from 8 studies (44.4%). Based on pooled data analysis, compared with clinicians, the OR for diagnostic error of AI models was 0.79 (95% CI, 0.48-1.31; *P* = .36; *I*^2^ = 60%) (Figure 1).

**Figure 2.**
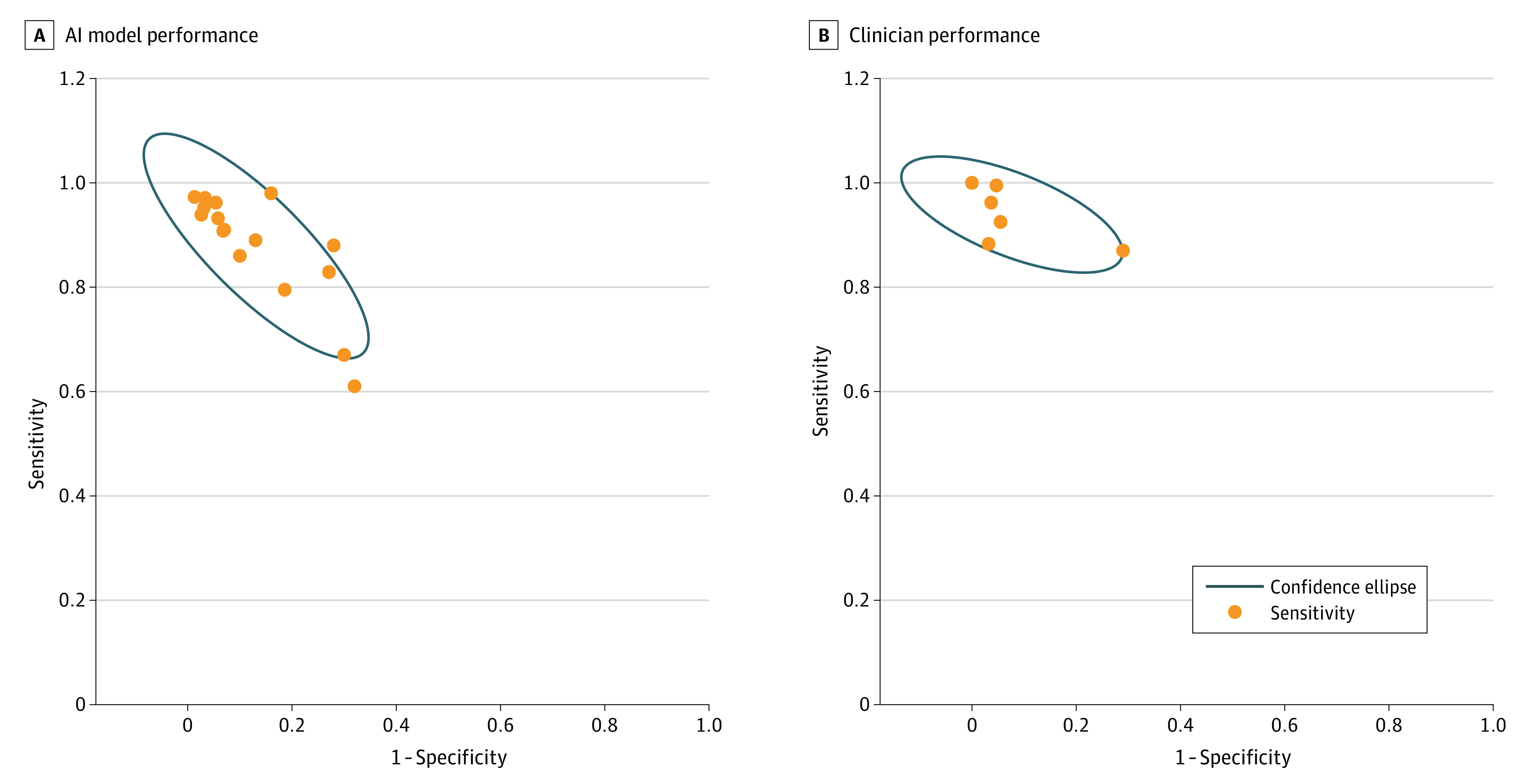
Sensitivity and Specificity of Artificial Intelligence (AI) Models Used for Diagnosing Hip Fractures on Radiographs and of Clinicians Who Used the Same Test Data Set

Among the included AI models, the mean (SD) sensitivity was 89.3% (8.5%), specificity was 87.5% (9.9%), and F1 score was 0.90 (0.06). Despite AI models having a higher overall accuracy, there was more variability in the sensitivity and specificity across models compared with clinician performance (Figure 2). Sensitivity ranged across studies from 67.0% to 98.0%, and specificity ranged from 70.0% to 98.7% (eTable 3 in Supplement 1). This wide range was predominantly due to 1 outlying study because 13 of 14 models (92.9%) reported a sensitivity greater than 80% and 11 of 14 models (78.6%) reported a specificity greater than 80%. eTable 3 in Supplement 1 also provides a breakdown of the F1 scores, κ scores, and Youden indexes of included AI models.

### Postoperative Outcome Prediction

Machine learning models have been developed to predict the outcome of 6 different postoperative outcomes following hip fracture surgery: mortality (15 studies^49,50,52,53,54,55,56,57,58,59,60,63,64,65,66,67^), length of stay (3 studies^48,51,62^), delirium (1 study^58^), discharge destination (1 study^47^), hospital cost (1 study^51^), 30-day major complications (1 study^64^), and functional independence measure (1 study^61^) (Table 2). Age (18 of 21 studies^47,49,50,51,52,54,55,56,57,58,59,60,61,63,64,65,66,67^ [85.7%]) and sex (17 of 21 studies [80.9%]^47,48,49,50,51,52,54,55,56,57,58,60,61,64,65,66,67^) were the most used features, whereas all other input features varied widely across studies^60^ and databases (eTable 4 in Supplement 1).

For 30-day mortality, median accuracy of the ML models was 72.8% (range, 71.0%-93.0%; n = 3), median AUC was 0.80 (range, 0.76-0.93; n = 6), median sensitivity was 73.0% (range, 68.0%-94.0%; n = 3), and median specificity was 72.8% (range, 61.0%-97.0%; n = 5). For the ML models, 1-year mortality median accuracy was 85.8% (range, 68.0%-95.0%; n = 3), median AUC was 0.81 (range, 0.72-0.99; n = 9), median sensitivity was 70.0% (range, 68.0%-74.0%; n = 3), and median specificity was 67.2% (range, 61.0%-99.0%; n = 3). In predicting length of stay, 1 ML model reported an AUC of 0.88 and an accuracy of 76.5%^51^; accuracy statistics were not available from the other 2 studies. In predicting hospital costs, accuracy was 79.0% and AUC was 0.89 (n = 1). The AUCs were 0.79 for predicting 30-day postoperative delirium (n = 1), 0.73 for discharge destination (n = 1), and 0.86 for functional independence measure (n = 1).

There were only enough studies to compare the difference in ML prediction to traditional statistical models (multivariable linear or logistic regression) for the postoperative mortality outcome. The mean AUC for ML models was 0.84 compared with 0.79 for alternative controls (*P* = .09) (eTable 5 in Supplement 1).

## Discussion

This study identified a mixed utility of AI models to assist in the management of patients presenting with hip fractures. Multiple studies have developed independent, reliable algorithms specifically to diagnose hip fractures based on routinely collected plain radiographic imaging (eFigure 2 in Supplement 1). In this study, the meta-analysis conducted to compare the accuracy of these ML models revealed that the models are comparable with the mean performance of expert clinicians at diagnosing hip fractures. Across all included studies,^29,30,31,32,33,34,35,36,37,38,39,40,41,42,43,44,45,46,47,48,49,50,51,52,53,54,55,56,57,58,59,60,61,62,63,64,65,66,67^ there was a wider range of sensitivity and specificity compared with clinician performance. However, the range was negatively skewed by 1 study that attempted to classify fractures into 3 different categories despite a relatively small training sample size. This review also compared the prediction of postoperative outcomes following hip fracture surgery of deep AI models compared with standard multivariable logistic or linear regression techniques (eFigure 2 in Supplement 1) and found no significant difference in AUC between the 2 statistical techniques in 60% of studies. Overall, these results must be interpreted with caution as described by the limitations below.

Because up to 10% of suspected hip fractures are not diagnosed on initial pelvic radiograph, techniques aiding the rapid and accurate identification of these fractures is imperative to enable expedient surgical management.^68^ Such techniques are particularly prudent for hip fracture management because surgical delays longer than 24 hours are associated with a 20% increased risk of 30-day mortality, twice the risk of medical complications, and greater length of stay and medical costs.^22,69^ Therefore, being able to automate the diagnosis and forecast outcomes for these patients can have significant benefits for patients and health care systems. However, despite the broad potential, the safety and reliability must be understood before the adoption of any new technology.^70^ Contemporary ML algorithms compare more interactions between the included predictors in the aim to improve the accuracy of outcome prediction. The 2 studies^49,50^ in which the ML models significantly outperformed traditional regression were both trained on relatively smaller data sets in the hundreds of training examples compared with thousands to tens of thousands. Studies that included larger sample sizes did not show similar advantages for ML. Nevertheless, because hip fractures are a common and expensive condition to manage, small improvements in prediction accuracy may help hospitals plan resources, such as staffing, beds, budget and implant procurement, improving system efficiency, and reducing expenses.

This study identified excellent potential in using AI to diagnosis hip fractures on routinely acquired plain radiographs; however, there are significant limitations and validation steps required before implementation. Missing a hip fracture (delaying diagnosis and possibly allowing fracture displacement) needs to be minimized by these algorithms (ie, avoiding false-negative results). Models are typically trained to maximize sensitivity and specificity by using an outcome metric that combines both (eg, AUC or F1 score). However, an alternative approach may be to train models to reduce false-negative results, followed by clinician screening of radiographs flagged as positive by the AI model. In the study with the largest training data set (approximately 5136 images), Kitamura^35^ trained an algorithm that had 86.0% sensitivity and 90.0% specificity. Although the algorithm was able to detect pelvic imaging position, hardware presence, and pelvic and acetabular fractures, it was most accurate at diagnosing proximal femoral fractures. To reduce errors, training should be performed using a larger number of outlier examples or oversampling techniques, such as random or borderline oversampling or adaptive synthetic sampling.^71^ A key consideration in the accuracy of hip fracture diagnoses was that although the performance of most ML models was comparable with the performance of expert radiologists and orthopedic surgeons, ML models consistently outperform trainees and nonexpert clinicians. This finding suggests that ML model use may be considered in assisting diagnoses in remote and resource-poor settings, where expert clinicians are not available.

Studies also demonstrated improvements in the accuracy of hip fracture diagnoses by experts when aided by ML algorithms. Considering that radiologists and orthopedic surgeons are facing increasing volumes of patients requiring radiographic interpretation for hip pathologies, addition of ML models in aiding expert review can accelerate image interpretation and decrease processing times. The British Orthopaedic Association guidelines on managing hip fractures states that patients presenting to hospitals for hip fractures should be transferred to the ward within 4 hours of presentation.^72^ This guideline has been shown to be a marker for quality of care provided by each hospital, and adherence to these guidelines has reduced time from admission to the operation room, reduced mortality at 30 days, and reduced mortality at 1 year.^73^ However, there are poor rates of adherence to these guidelines. A potential solution is incorporation of ML models in clinical practice to accelerate diagnosis, prognosis determination, and admission planning, which may ultimately decrease time to transfer to ward and time to the operating room.^46^

All generated algorithms have been studied on retrospective data and evaluated using a holdout sample. It is also essential to perform further external validation at institutions separate from where the algorithms were trained, which no included studies performed. Across all specialties, 75% of algorithms perform moderately or substantially worse during external validation,^74^ which may be due to different methods of data collection, imaging scanners, resolution, formatting, or image capture protocols. In addition, algorithms must also be studied on prospective data in a native clinical environment before their regular use. Integrating this technology into routine care pathways will allow the algorithms to be evaluated not only on accuracy but also on other important criteria, such as impact on patient outcomes, user acceptance, efficiency, resource utilization and planning, computational time, and cost. Integration into practice also comes with practical challenges, such as acceptability and ethical and legal responsibilities. Algorithms that provide a confidence level of their decision can be used to help aid clinician decision-making and patient screening to improve the efficiency and reduce the burden of work for clinicians.

### Limitations

To our knowledge, this study was the first evaluation of the literature surrounding AI algorithms developed for the management of hip fractures from diagnosis to postoperative outcomes. This review provides insight into the potential impact that applying AI has on the management of one of the most common, resource-intensive, and devastating diagnoses. Nonetheless, there are limitations to the algorithms and methods of included studies, as mentioned previously, as well as to our review. A hierarchal summary receiver operating curve summarizing performance across all studies was unable to be created because studies did not report data in the form of a contingency table. Various AI techniques and predictive features were used across all studies, but we were unable to compare the use of each strategy and the effect this had on algorithm performance due to data and study reporting heterogeneity. Additionally, the quality of studies was unable to be properly evaluated because the Transparent Reporting of a Multivariable Prediction Model for Individual Prognosis or Diagnosis for Artificial Intelligence (TRIPOD-AI) guidelines are still under development.^75^

## Conclusions

This systematic review and meta-analysis helps evaluate the literature surrounding the current development of AI applications for the management of hip fractures. The potential applications regarding the use of AI to aid with diagnosis from hip and pelvic radiographs are promising. However, the use of AI does not seem to provide substantial additional benefit over traditional multivariable predictive statistics. The results of these applications are variable, which may be due to the quality or quantity of data from which these algorithms are developed rather than a true limitation of AI’s power. Further studies should focus on evaluating whether these limitations remain with the use of large, accurate, multi-institutional data sets. Moreover, studies externally validating and implementing hip fracture diagnostic algorithms need to be performed to assess the effect on patient care.

## Notes

Supplement 1. **eFigure 1.** PRISMA Flowchart of Included Studies **eFigure 2.** Depiction of Typical Machine Learning Models Used for Diagnosing Hip Fractures From Medical Imaging, Representing a Convolutional Neural Network (A), and for Predicting Postoperative Patient Clinical Outcomes From a Multilayer Perceptron (B) **eTable 1.** Machine Learning Algorithm Properties of Studies on Hip Fracture Diagnosis Using Plain Radiographs **eTable 2.** Databases Used for Outcome Prediction With Availability **eTable 3.** Summary of Outcome Performance of Artificial Intelligence Models for Diagnosing Hip Fractures Based on Plain-Film Radiographs **eTable 4.** Common Features Used for Postoperative Outcome Prediction by Each Study **eTable 5.** Comparison of Area Under the Curve Values Between Machine Learning Models and Traditional Statistical (Multivariable Logistic or Linear Regression) Prediction Models

Supplement 2.
Data Sharing Statement
