# Supplementary material for: Artificial Intelligence for Hip Fracture Detection and Outcome Prediction: A Systematic Review and Meta-analysis
Source: JAMA Netw Open. 2023 Mar 17;6(3):e233391. doi: 10.1001/jamanetworkopen.2023.3391 (PMC10024206; doi:10.1001/jamanetworkopen.2023.3391)
Supplement: Supplement 1. — eFigure 1. PRISMA Flowchart of Included Studies eFigure 2. Depiction of Typical Machine Learning Models Used for Diagnosing Hip Fractures From Medical Imaging, Representing a Convolutional Neural Network (A), and for Predicting Postoperative Patient Clinical Outcomes From a Multilayer Perceptron (B) eTable 1. Machine Learning Algorithm Properties of Studies on Hip Fracture Diagnosis Using Plain Radiographs eTable 2. Databases Used for Outcome Prediction With Availability eTable 3. Summary of Outcome Performance of Artificial Intelligence Models for Diagnosing Hip Fractures Based on Plain-Film Radiographs eTable 4. Common Features Used for Postoperative Outcome Prediction by Each Study eTable 5. Comparison of Area Under the Curve Values Between Machine Learning Models and Traditional Statistical (Multivariable Logistic or Linear Regression) Prediction Models [file jamanetwopen-e233391-s001.pdf]

Supplementary Online Content

Lex JR, Di Michele J, Koucheki R, Pincus D, Whyne C, Ravi B. Artificial intelligence for hip fracture detection and outcome prediction: a systematic review and meta-analysis. *JAMA Netw Open*. 2023;6(3):e233391. doi:10.1001/jamanetworkopen.2023.3391

eFigure 1. PRISMA Flowchart of Included Studies

eFigure 2. Depiction of Typical Machine Learning Models Used for Diagnosing Hip Fractures From Medical Imaging, Representing a Convolutional Neural Network (A), and for Predicting Postoperative Patient Clinical Outcomes From a Multilayer Perceptron (B)

eTable 1. Machine Learning Algorithm Properties of Studies on Hip Fracture Diagnosis Using Plain Radiographs

eTable 2. Databases Used for Outcome Prediction With Availability

eTable 3. Summary of Outcome Performance of Artificial Intelligence Models for Diagnosing Hip Fractures Based on Plain-Film Radiographs

eTable 4. Common Features Used for Postoperative Outcome Prediction by Each Study

eTable 5. Comparison of Area Under the Curve Values Between Machine Learning Models and Traditional Statistical (multivariable logistic or linear regression) Prediction Models

This supplementary material has been provided by the authors to give readers additional information about their work.

eFigure 1 – PRISMA flow chart of included studies.

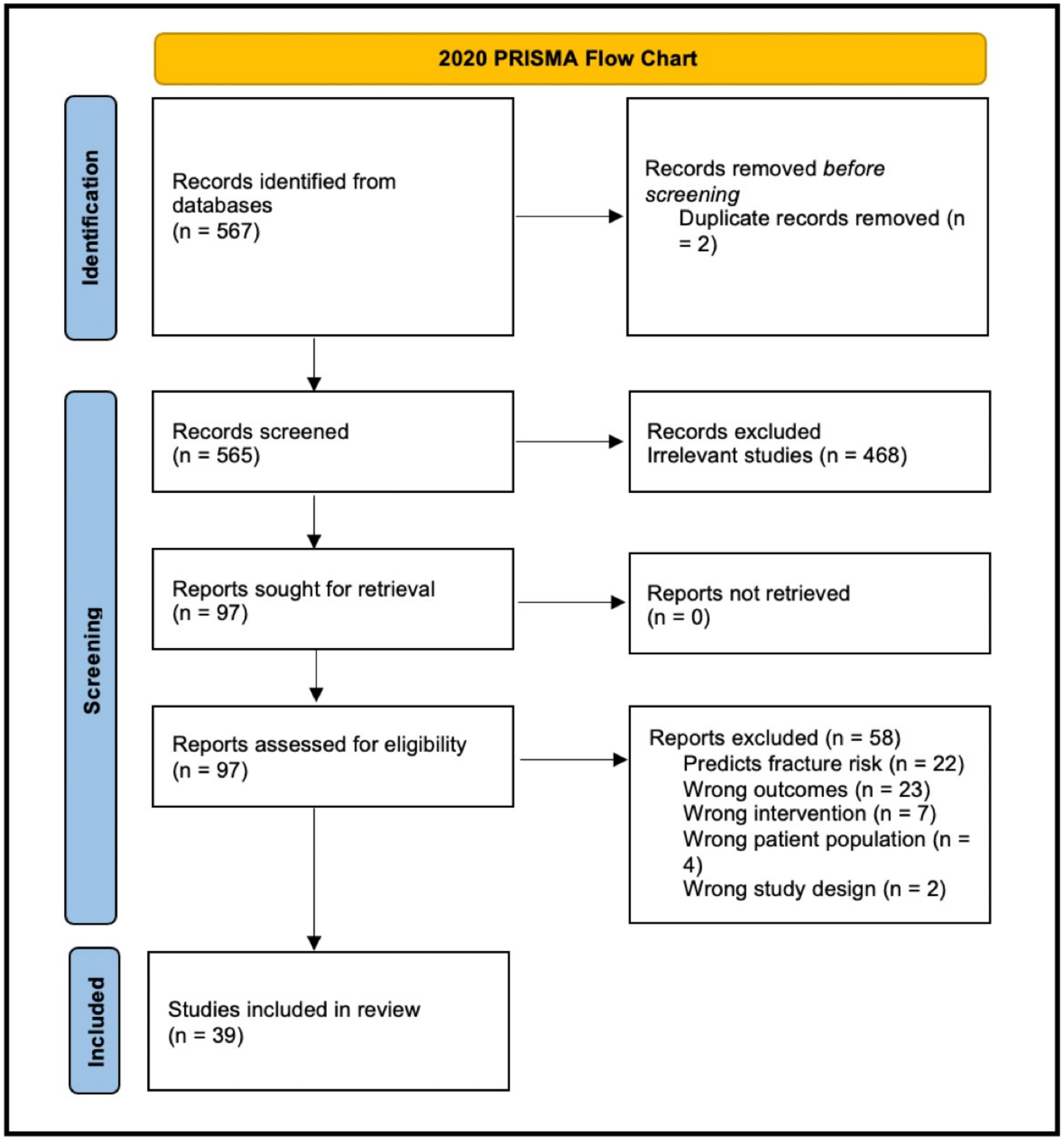

© 2023 Lex JR et al. *JAMA Network Open.*

eFigure 2 – Depiction of typical machine learning models used for diagnosing hip fractures from medical imaging, representing a convolutional neural network (A), and for predicting post-operative patient clinical outcomes from a multilayer perceptron (B).

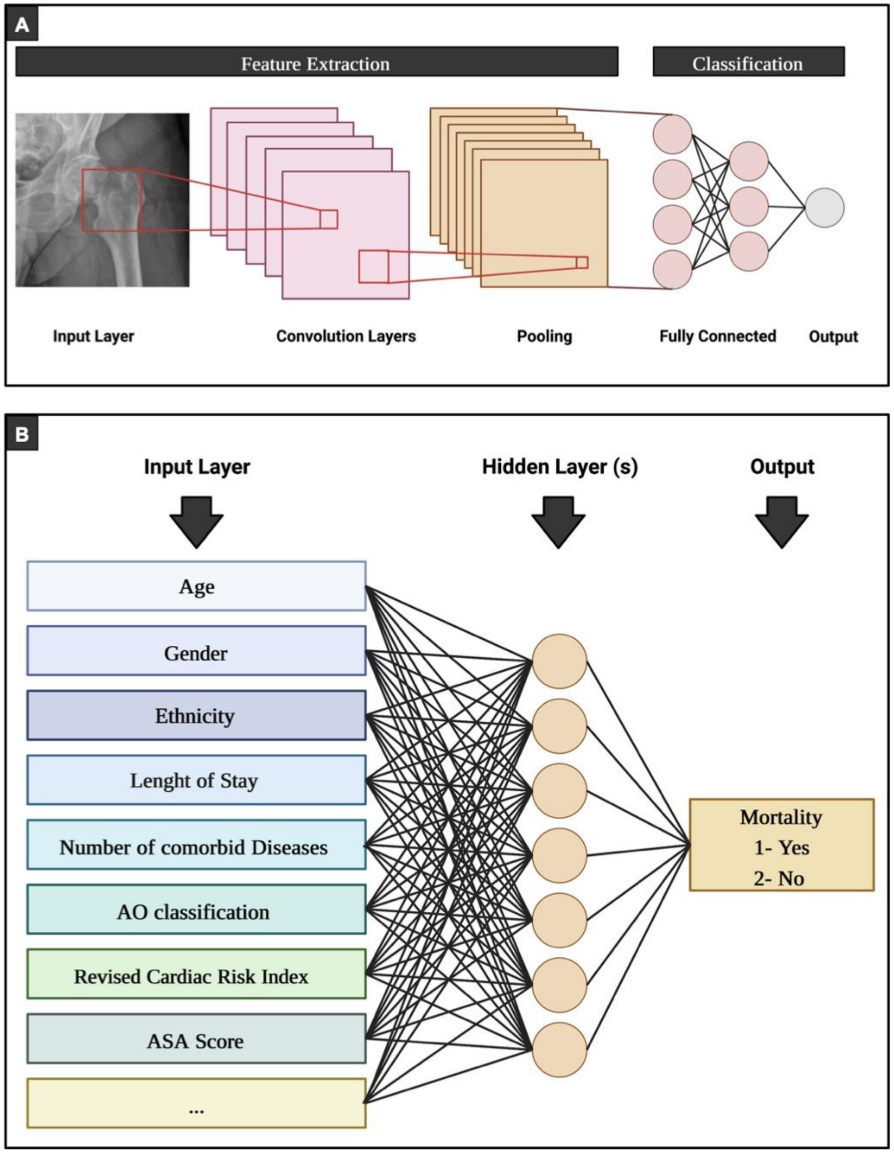

© 2023 Lex JR et al. *JAMA Network Open.*

eTable 1 – Machine learning algorithm properties of studies on hip fracture diagnosis using plain radiographs.

FCV = Fold cross validation

Study	# Epochs used to train the Model Trained	Was Augmentation utilized?	# Fold Cross Validation
**Cheng, 2019[29]**	60	Yes	-
**Urakawa, 2019[30]**	-	Yes	-
**Adams, 2019[31]**	50	Yes	-
**Mawatari, 2020[32]**	-	Yes	-
**Jimenez-Sanchez, 2020[33]**	80 or 200	Yes	-
**Yu, 2020[34]**	200	Yes	20-FCV
**Kitamura, 2020[35]**	400	Yes	-
**Krogue, 2020[36]**	10	Yes	-
**Yamada, 2020[37]**	100	Yes	-
**Mutasa, 2020[38]**	200	Yes	-
**Beyaz, 2020[39]**	50	Yes	5-FCV
**Acici 2021[40]**	50	No	5-FCV
**Bae, 2021[41]**	-	Yes	-
**Cheng, 2021[42]**	100	Yes	5-FCV
**Guy, 2021[43]**	-	Yes	8-FCV
**Twinprai, 2022[44]**	60	Yes	-
**Murphy, 2022[45]**	-	Yes	-
**Liu, 2022[46]**	-	No	-

© 2023 Lex JR et al. *JAMA Network Open.*

eTable 2– Databases used for outcome prediction with availability.

Study	Database	Data Availability
**Ottenbacher, 2004**[47]	1- Uniform Data System for Medical Rehabilitation (UDSMR)<br>2- National Follow-up Services (NFS)	Private. Available for purchase.
**Sund, 2009**[48]	Finnish Health Care Register	Available with Permission.
**Lin, 2010**[49]	Department of Orthopedics, National Taiwan University Hospital Yun-Lin Branch Database	Hospital owned.
**Shi, 2013**[50]	Department of Orthopedics, First Affiliated Hospital, Liaoning Medical University Database	Hospital owned.
**Karnuta, 2019**[51]	New York Statewide Planning and Research Cooperative System Database	Available with Permission.
**Chen, 2020**[52]	National Health Insurance Research Database - Taiwan	Available with Permission.
**DeBaun, 2020**[53]	National Surgical Quality Improvement Program	Available with Permission.
**Zhang, 2020**[54]	A hospital-owned patient management database	Hospital owned.
**Cao, 2021**[55]	Swedish National Quality Registry	Available with Permission.
**Cowling, 2021**[56]	National datasets of routinely collected hospital data and official death records from England	Available with Permission.
**Forssten, 2021**[57]	Swedish National Quality Registry	Available with Permission.
**Oosterhoff, 2021**[58]	National Surgical Quality Improvement Program	Available with Permission.
**Li, 2021**[59]	Chinese PLA General Hospital Hip Fracture Study Database	Hospital owned.
**Cary Jr, 2021**[60]	1- Inpatient Rehabilitation Facility–Patient Assessment Instrument (IRF-PAI)<br>2- The Medicare Provider Analysis and Review (MedPAR)<br>3- Master Beneficiary Summary Files	Available for purchase.
**Shtar, 2021**[61]	Petach Tikva, Israel Geriatric Rehabilitation Center Database	Hospital owned.
**Zhong, 2021**[62]	Tianjin Medical University General Hospital Database	Hospital owned.
**Xing, 2022**[63]	West China Hospital	Hospital owned.
**Harris, 2022**[64]	National Surgical Quality Improvement Program	Available with Permission.
**Oosterhoff, 2022**[65]	Multiple hospitals in the USA	Hospital owned.
**Lei, 2022**[66]	Medical Information Mart for Intensive Care-III	Available with Permission.
**Kitcharanant, 2022**[67]	Siriraj Hospital Database	Hospital owned.

© 2023 Lex JR et al. *JAMA Network Open.*

eTable 3 – Summary of outcome performance of artificial intelligence models for diagnosing hip fractures based on plain-film radiographs.

Study	Sensitivity	Specificity	F1-score	Kappa Score	Youden Index
**Cheng, 2019[29]**	98%	84%	0.916	-	0.82
**Urakawa, 2019[30]**	93.90%	97.4%	-	-	0.91
**Adams, 2019[31]**	-	-	-	-	-
**Mawatari, 2020[32]**	88%	72%	-	-	0.60
**Jimenez-Sanchez, 2020[33]**	-	-	0.94	-	-
**Yu, 2020[34]**	97.10%	96.70%	-	-	0.94
**Kitamura, 2020[35]**	86.0%	90.0%	-	-	0.76
	-	-	-	-	-
**Krogue, 2020[36]**	-	-	-	-	-
	93.20%	94.20%	-	-	0.87
**Yamada, 2020[37]**	-	-	0.98	-	-
**Mutasa, 2020[38]**	91%	93%	-	-	0.84
**Beyaz, 2020[39]**	82.9%	72.9%	0.836	0.554	0.56
**Acici 2021[40]**	79.5%	81.4%	0.803	0.609	0.61
**Bae, 2021[41]**	97.3%	98.7%	-	-	0.96
**Cheng, 2021[42]**	90.8%	93.2%	-	-	0.84
**Guy, 2021[43]**	67%	70%	-	-	0.37
**Twinprai, 2022[44]**	96.2%	94.6%	0.909	-	0.91
**Murphy, 2022[45]**	-	-	0.91	0.87	-
**Liu, 2022[46]**	89.0%	87.0%	-	-	0.76

© 2023 Lex JR et al. *JAMA Network Open.*

eTable 4 – Common features used for post-operative outcome prediction by each study.
LOS = Length of stay; ASA = American Society of Anaesthesiologists Score; CCI = Charlson Comorbidity Index; RCRI = Revised Cardiac Risk Index; DM = Diabetes mellitus; HTN = Hypertension; OA = Osteoarthritis; CVD = Cardiovascular disease; CHF = Congestive heart failure; CVA = Cerebrovascular accident; COPD = Chronic obstructive pulmonary disease; PNA = Pneumonia; CKD = Chronic kidney disease; SES = Socioeconomic status.

Study	Outcome Predicted	Fracture Type / Fracture pattern	Age	Sex	Ethnicity / Race	LOS	ASA	CCI score	RCRI score	DM	HTN	OA	CVD / CHF	CVA	COPD	PNA	CKD / Dialysis	Anemia / Hb	Dementia / Cognitive impairment	Cancer	SES	Functional status
**Lin, 2010[49]**	Mortality	✓	✓	✓			✓			✓	✓		✓	✓					✓			
**Shi, 2013[50]**	Mortality		✓	✓						✓			✓						✓	✓		
**Chen, 2020[52]**	Mortality	✓	✓	✓																	✓	
**DeBaun, 2020[53]**	Mortality												✓		✓	✓	✓			✓		
**Zhang, 2020[54]**	Mortality	✓	✓	✓			✓											✓				
**Cao, 2021[55]**	Mortality		✓	✓			✓		✓		✓								✓			
**Cowling, 2021[56]**	Mortality		✓	✓																	✓	
**Forssten, 2021[57]**	Mortality		✓	✓			✓	✓	✓	✓	✓		✓	✓	✓		✓		✓	✓		
**Li, 2021[59]**	Mortality		✓			✓					✓					✓	✓					
**Cary Jr, 2021[60]**	Mortality		✓	✓	✓			✓		✓				✓	✓	✓	✓					✓
**Xing, 2022[63]**	Mortality		✓												✓			✓		✓		
**Oosterhoff, 2022[65]**	Mortality		✓	✓									✓	✓	✓			✓				
**Lei, 2022[66]**	Mortality		✓	✓									✓		✓			✓				
**Kitcharanant, 2022[67]**	Mortality		✓	✓				✓					✓	✓	✓		✓					
**Harris, 2022[64]**	Mortality, Major Complications		✓	✓							✓		✓		✓		✓			✓		
**Karnuta, 2019[51]**	LOS		✓	✓	✓			✓														
**Sund, 2009[48]**	LOS			✓		✓				✓		✓	✓	✓	✓				✓	✓		
**Zhong, 2021[62]**	LOS							✓														
**Ottenbacher, 2004[47]**	Living setting		✓	✓	✓	✓															✓	✓
**Shtar, 2021[61]**	mFIM	✓	✓	✓														✓	✓			✓
**Oosterhoff, 2021[58]**	Post-op Delirium		✓	✓															✓			✓

© 2023 Lex JR et al. *JAMA Network Open.*

eTable 5 – Comparison of Area Under the Curve values between machine learning models and traditional statistical (multivariable logistic or linear regression) prediction models.
ML = Machine learning; AUC = Area Under the Curve

Study	ML Model 1 year mortality AUC	Control 1 year mortality AUC
Lin, 2010[49]	0.949	0.784
Shi, 2013[50]	0.901	0.745
Cowling, 2021[51]	0.804	0.798
Forssten, 2021[52]	0.72	0.74
Cary Jr, 2021[60]	0.758	0.756
Xing, 2022[63]	0.813	0.797
Kitcharanant, 2022[67]	0.95	0.91
**Average**	**0.84**	**0.79**
**P-value**	**P = 0.09**	

© 2023 Lex JR et al. *JAMA Network Open.*
