# Supplementary material for: Artificial Intelligence for Hip Fracture Detection and Outcome Prediction: A Systematic Review and Meta-analysis
Source: JAMA Netw Open. 2023 Mar 17;6(3):e233391. doi: 10.1001/jamanetworkopen.2023.3391 (PMC10024206; doi:10.1001/jamanetworkopen.2023.3391)
Supplement: Supplement 2. — Data Sharing Statement [file jamanetwopen-e233391-s002.pdf]

Data Sharing Statement

Lex. Artificial Intelligence for Hip Fracture Detection and Outcome Prediction. *JAMA Netw Open*. Published March 17, 2023. doi:10.1001/jamanetworkopen.2023.3391

Data
Data available: No
